# Fistule vésico-utérine

**DOI:** 10.11604/pamj.2014.18.94.4401

**Published:** 2014-05-27

**Authors:** Mehdi Kehila, Rim Ben Hmid

**Affiliations:** 1 Faculté de médecine de Tunis, Service C du Centre de Maternité et de Néonatologie, Tunis, Tunisie

**Keywords:** Fistule vésico-utérine, hématurie, cystoscopie, Vesicouterine fistulas, hematuria, cystoscopy

## Image en medicine

Patiente âgée de 32 ans qui a consulté pour une aménorrhée secondaire de 6 mois associée à une hématurie cyclique. Elle est 2^ème^ geste, 2^ème^ pare. Dans ses antécédents on note un premier accouchement par césarienne pour une souffrance foetale. La deuxième grossesse s'est compliquée d'un hématome rétro-placentaire à 37 semaines d'aménorrhée secondaire à un accident de la voie publique. La patiente a eu alors une césarienne en urgence dans un contexte de troubles d'hémostase. Elle a séjourné par la suite pendant 15 jours en réanimation. Elle n'a pas eu de retour de couches mais des hématuries cycliques. Elle a eu une cystoscopie qui a montré un orifice fistuleux au niveau de la face postérieure de la vessie. On a complété par une urographie intraveineuse qui a montré une fistule utéro-vésicale. La patiente a eu une cure chirurgicale par voie haute de la fistule avec bonne évolution: disparition de l'hématurie et retour à un cycle normal.

**Figure 1:**
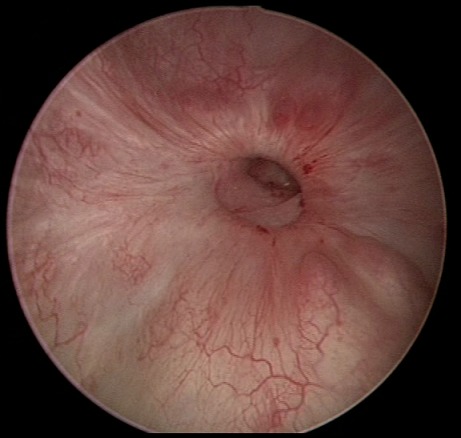
Photo cystoscopique de l'orifice vésical de la fistule

